# Genomics of Adaptation and Speciation

**DOI:** 10.3390/genes13071187

**Published:** 2022-07-01

**Authors:** Walter W. Wolfsberger, Fabia U. Battistuzzi, Taras K. Oleksyk

**Affiliations:** 1 Department of Biological Sciences, Oakland University, Rochester, MI 48307, USA; wwolfsberger@oakland.edu (W.W.W.); battistu@oakland.edu (F.U.B.); 2 Department of Biology, Uzhhorod National University, 88000 Uzhhorod, Ukraine; 3 Biology Department, University of Puerto Rico at Mayagüez, Mayagüez 00682, Puerto Rico

The availability of genome data provides a unique window into speciation mechanisms with virtually infinite amounts of information, providing a pathway for a better understanding of major evolutionary questions. The process of speciation is a key aspect of understanding biodiversity. Each species inhabits a specific environment, a niche, that, through the action of natural selection and other evolutionary forces, shapes its unique genomic program–genome sequence, its diversity and expression. Species exist as genetic pools within a specific area or ecological niche, as variations of constantly recombining genetic code, and they diverge when the exchange of information between populations is interrupted. As environments continuously change, some species can change and adapt, while others, lacking genome variation, cannot keep up with the change and become extinct. Genetic diversity is continuously formed by a balance of mutation and drift, contributing to diversity across the genome and creating a backdrop for the action of natural selection, enabling adaptation [1,2] (Ellegren and Galtier, 2016; Oleksyk et al., 2010).

Recent computational developments are supplying unprecedented power to simulations, as well as analytic and reconstruction algorithms, that help uncover factors that define the genetic potential of populations and species. Adaptability is commonly defined in terms of existing genetic variation within each species, and it is generally accepted that preserving genetic diversity is required for adaptability: in other words, a species that has lost all its reserves of genetic diversity is doomed to extinction [3,4,5,6] (Jones et al., 2012; Lai et al., 2019; Reid et al., 2016; Visser, 2008). Genome data from natural populations provide us with the means to evaluate past and present genetic diversity, providing a new way to develop effective conservation strategies [7,8] (Mable, 2019; Hoelzel et al., 2019). In this Editorial, we review and classify examples of different approaches to studying adaptation and speciation in natural populations using genome-scale data from the collection of articles in this Special Issue.

## 1. Reproductive Barriers and Reinforcement Are Better Understood from the Whole-Genome Perspective

The large structural differences between genomes may not even require sequencing or genotyping and can be studied with more traditional technologies. For instance, post-zygotic reproductive isolation is often linked to chromosomal differences and keeps species isolated and evolving independently, even with the wide geographical contact of habitats. A study of chromosomal polymorphisms as a reproductive barrier and speciation mechanism for both inter- and intraspecific hybrids in the *Mazama* genus demonstrated the possibility of interspecific hybrids that were viable until maturity but presumably infertile, while the intraspecific hybrids showed a low negative effect on reproductive fitness [9] (Galindo et al., 2021). In another example, a case of hybridogenesis was demonstrated through showing genome introgressions and elimination in edible frog *Pelophylax kl. esculentus* (RL), a natural hybrid between the marsh frog *P. ridibundus* (RR) and the pool frog *P. lessonae* (LL) [10] (Miura et al., 2021).

## 2. Genomic Data Provide Us with the Ability to Resolve Questions about Speciation at the Level of Phylogenetic Relationships

Genome data were used to investigate times of speciation in the context of the island colonization of the Greater Antilles by a group of Amazon parrots [11,12] (Kolchanova et al., 2021, 2019). A collection of parrot genomes was aligned to reconstruct phylogenetic relationships and estimate the times of speciation, providing several hypotheses based on a combination of molecular data and biogeographic modeling to understand the history of speciation of the parrots from the Caribbean islands. In another example, major structural rearrangements were evaluated in the context of speciation in highly conserved genomes of seals (*Pinnipedia*) [13] (Beklemisheva et al., 2020). The karyotypic divergence of conserved genomes of pinnipeds occurred largely at the level of heterochromatin or via intrachromosomal repositioning, and multiple interchromosomal rearrangements were detected and described in ten karyotypes.

## 3. Transcriptomic Sequences Help Us to Understand Species-Specific Phenotypes

Transcriptome sequencing has been demonstrated as useful not only in the context of describing environmental variation in gene expression, expression levels of the same gene may also contribute to species-specific phenotypes, making each species unique. A study in this collection shows that formation and loss of transcription factors (TFs) can enable unique expression profiles and provide variable switches that categorize divergent expression profiles into species-specific phenotypes [14] (Zhao and Kishino, 2020). While conserved TFs likely form the backbone structure of the regulatory network, the variable isolated TFs tune the flow of the regulatory network and give rise to species uniqueness by acting as on/off switches.

## 4. Genomic Approaches Provide a New Way of Understanding the Role of Variation in Adaptation

Genome-wide data allow the visualization and interpretation of genome variation in the context of evolutionary processes driven by natural selection, migration, mutation, and drift. Genomic variation provides an important key to understanding the mechanisms of evolution in the context of previous environmental conditions. In one of the studies from this issue, genomic sequence data from Douglas-fir, *Pseudotsuga menziesii* trees combined with genotypes of several hundred seeds, as well as multivariate scores of environmental variables and cold-related traits, were used to evaluate the molecular basis of cold adaptation [15] (de La Torre et al., 2021). Associations with cold adaptation were identified in 130 genes involved in many important biological functions related to growth, phenology, and cold hardiness, and exhibited variation in response to changing environments, providing a baseline for studies of the phenotypic variation of species under climate change. In another study, genome data of the Caspian whipsnake (*Dolichophis caspius*), currently experiencing geographical range isolation and local endangerment, were used to evaluate its adaptive potential, as well as its structure, admixture, and migration patterns [16] (Mahtani-Williams et al., 2020). Specific genotypes in hundreds of genes were found to be associated with the distribution and adaptation of *D. caspius* and correlated with seven climatic variables (such as isothermality, annual habitat range, and annual mean temperature).

## 5. Genome-Wide Visualization of Genetic Diversity Helps Us to Understand Limitations of Adaptability in Endangered Species

Ultimately, bioinformatics approaches create new opportunities to evaluate the signatures of evolutionary forces in species to understand what happens to genetic variation when they are born, adapt and die. Generally, the low heterozygosity identified from genome data points to high levels of inbreeding, non-random mating, population fragmentation, and potential recent bottlenecks. Generally, genetic diversity is usually estimated as heterozygosity across neutral markers [11,17] (Kirk and Freeland, 2011; Nei, 1978). Heterozygosity has been routinely used to evaluate the genetic potential of a population faced with extinction, and a significant majority of threatened taxa show lower genetic diversity than taxonomically related, but not threatened, taxa [18] (Spielman et al., 2004). However, heterozygosity across neutral markers may not be directly related to conservation, since no simple general relationship exists between neutral genetic diversity measured as overall heterozygosity and the risk of species extinction. Instead, variation in functional loci, recent demographic history, and ecological relationships have been proposed for evaluating risk via effective conservation genetic strategies [19] (Teixeira and Huber, 2021). Therefore, in the case of an endangered species, the loss of genetic variation that directly impacts fitness is more appropriate [7,11,20] (Amos and Balmford, 2001; Kirk and Freeland, 2011; Mable, 2019), and genetic data collected across a limited number of neutral loci have limited use.

By visualizing genome diversity among high-quality genome assemblies, Totikov et al. (2021) [21] demonstrated how technological breakthroughs in genomics could provide conservation biology with valuable new approaches. Chromosome maps can also be used to investigate the distribution of genetic diversity in different parts of genomes of endangered and threatened species. In the future, chromosome-level assemblies could provide a well-justified pathway for future genome-wide studies to access the adaptability of an endangered species. In other words, while traditional genetic methods may still provide some answers to conservation questions [22] (McMahon et al., 2014), new methods based on high-quality assemblies give way to a completely new level of opportunities, which can provide information focusing on the genetic diversity underlying adaptability and the rebound potential of an endangered species.

## 6. Summary

Genomics is becoming increasingly important for solving the major evolutionary questions about adaptation and speciation. Genetic diversity within a species is valuable, not only due to the existence of combinations of genes and alleles present at a given time in a population, but also because it contributes to ongoing evolutionary processes [23] (Stange et al., 2021). Answering questions about speciation, adaptation, and extinction does not require the newest and most advanced sequencing technologies, and limited sets of data can be used. The examples in this issue included studies of large structural and chromosomal variation [9,13] (Beklemisheva et al., 2020; Galindo et al., 2021), mtDNA and/or limited nuclear marker approaches to phylogenetics [24] (Kolchanova et al., 2021), hybridogenesis [10] (Miura et al., 2021), comparative transcriptomics [14] (Zhao and Kishino, 2020), and genome-wide studies of SNP variation using a combination of low-coverage sequencing and genotyping [15,16] (de La Torre et al., 2021; Mahtani-Williams et al., 2020). However, newer, more contiguous assemblies may allow for a better estimation of the genetic diversity, localization, and visualization of the distribution of genome diversity in the evolutionary context of adaptation and speciation [21] (Totikov et al., 2021).

In this Special Issue, we presented examples of different approaches to studying adaptation and speciation using genome-scale data. Genome-scale data allow us to resolve patterns of phylogenetic relationships and species evolution, identify reproductive barriers, and reinforce the speciation process. Transcriptomic data are complementary in the context of variable gene expression driven by environmental variation, which produces expression profiles that make species unique. Genomic approaches that incorporate sequencing and bioinformatics can identify signatures of adaptation, and more contiguous assemblies may allow for better estimates. Finally, technological breakthroughs in genomics allow us to understand and visualize genetic diversity among the chromosomes of endangered species.
